# Internal and Marginal Adaptation of Adhesive Resin Cements Used for Luting Inlay Restorations: An In Vitro Micro-CT Study

**DOI:** 10.3390/ma15176161

**Published:** 2022-09-05

**Authors:** Linah M. Ashy, Hanadi Marghalani

**Affiliations:** 1 DRBBA-Research Group, Oral and Maxillofacial Prosthodontics Department (OMP), Faculty of Dentistry, King Abdulaziz University (KAU), Jeddah 21589, Saudi Arabia; 2 DRBBA-Research Group, Operative and Esthetic Dentistry Division, Restorative Dentistry Department (RDD), Faculty of Dentistry, King Abdulaziz University (KAU), Jeddah 21589, Saudi Arabia

**Keywords:** resin cements, ceramic inlays, X-ray microtomography, marginal and internal adaptation

## Abstract

Adequate internal adaptation and marginal sealing of resin luting cements are of particular importance for the success of cemented ceramic inlays. The purpose of this study was to investigate the initial adaptation of different resin cements at the tooth-inlay restoration interface at enamel versus dentin surfaces. Thirty-two extracted human molars were allocated to four groups. One Class II cavity was prepared in each tooth. In each group, half of the cavities’ gingival floors were on enamel while the other halves were on cementum. Lava Ultimate CAD/CAM inlays were luted to the cavities using the following adhesive systems: RelyX Unicem, RelyX Ultimate, *e*Cement, and Variolink Esthetic DC. After staining teeth with silver nitrate solution, marginal and internal gap volumes were determined using micro-CT images. Statistical analyses were conducted by independent *t* test and one-way ANOVA followed by post hoc Tukey test (*p* < 0.05). The internal and marginal gap volume values were the highest for Variolink Esthetic DC at the dentin surface (0.629 ± 0.363) and (2.519 ± 1.007), respectively, and the lowest for RelyX Unicem at the enamel surface (0.005 ± 0.004) and (0.009 ± 0.003), respectively. The internal and marginal adaptation on the enamel surface for RelyX Unicem and RelyX Ultimate resin cements were comparable to each other and to *e*Cement but significantly better than Variolink Esthetic DC cement. Regardless of the adhesive resin system used, adaptation on enamel is superior to that on dentin surfaces.

## 1. Introduction

All ceramic restorations have demonstrated superior optical and mechanical properties as well as a clinically acceptable marginal fit in comparison to porcelain-fused-to-metal crowns [1,2]. Adhesive luting resin cements are of particular importance for all ceramic inlays in order to obtain an acceptable micromechanical bonding to enamel and dentin. These cements have shown to provide enduring retention, increased resistance to fracture, and maintenance of a good marginal seal for all ceramic restorations [3,4]. Inadequate internal adaptation and marginal sealing of adhesive resin cements can lead to reduced strength and detrimental microleakage at the restoration margin [5]. The reduced sealing ability of adhesives was presumed to result from the high viscosity of the bonding agent decreasing its infiltration into microretention gaps [6]. Ghazy et al. reported inferior marginal sealing ability for the self-adhesive resin cement RelyX Unicem (3M ESPE, St. Paul, MN, USA) as compared to the cement with a two-step self-etch adhesive system Panavia F 2.0 (Kuraray, Tokyo, Japan) [7]. Haralur compared interfacial microleakage associated with four different types of resin cements: Variolink veneer (Ivoclar Vivadent, Schann, Liechtenstein), Panavia F 2.0 (Kuraray, Tokyo, Japan), RelyX ARC (3M ESPE, St. Paul, MN, USA), and RelyX Unicem (3M ESPE, St. Paul, MN, USA). He concluded the least microleakage with the etch-wash resin cement and the highest microleakage for the self-adhesive resin cement [8]. Previously, Tay and Pashley speculated that the reduced sealing ability of self-adhesive resin cements could be due to the transduction of dentinal fluids into the cement layer hindering its complete polymerization [9]. However, comparable marginal adaptation of self-adhesive resin cements and self-etch resin cements was reported in the literature [10]. Moreover, differences in the sealing ability among different self-adhesive resin cements were investigated [11,12]. Elbadrawy et al. found comparable results with regard to marginal seal for RelyX Unicem (3M ESPE, St. Paul, MN, USA) and Calibra (Dentsply, Charlotte, NC, USA) cements that were significantly higher than BisCem (Bisco, Schaumburg, IL, USA) [11]. Additionally, Uludag et al. reported lower microleakage at RelyX Unicem (3M ESPE, St. Paul, MN, USA) interface than with Smartcem 2 (Dentsply, Charlotte, NC, USA) and SpeedCem (Ivoclar Vivadent, Schann, Liechtenstein) resin cements [12]. Numerous studies have investigated the adaptation of adhesive luting cements for crowns and veneers [13,14,15]. However, the evaluation of different adhesive resin cements in association with inlay restorations is scarce and, to our knowledge, there are no reports in the literature on the adaptation of adhesive resin cements for inlays using micro-CT evaluation methods. Therefore, the purpose of this study was to investigate the internal and marginal adaptation/gap of four different resin cements at a tooth-inlay restoration interface on enamel and dentin surfaces. The null hypotheses tested were:

1. There is no difference in the internal and marginal adaptation of different types of adhesive resin cements.
2. There is no difference in the adaptation of adhesive resin cements on enamel versus dentin.

## 2. Materials and Methods

Research methodology was approved by the Research Ethics Committee at King Abdulaziz University Faculty of Dentistry with ethical approval # 048-04-17.

### 2.1. Samples Preparation

Thirty-two human molar teeth with approximately 10 mm mesiodistal dimensions were included in this study. Teeth were freshly extracted after obtaining informed consent. The teeth were debrided from soft tissue and hard calculus, then cleaned for 5 min with an ultrasonic scaler, and half of their roots were cut by a low-speed diamond saw (ISOMET Buehler Ltd., Lake Bluff, IL, USA). Samples were subsequently stored in distilled water at room temperature.

### 2.2. Cavity Preparation

One Class II cavity was prepared in each tooth by a single operator using a high-speed hand piece with a cylindrical flat-end diamond bur (Brasseler, Savannah, GA, USA) under water cooling. The dimensions of each proximal cavity were approximately 4 mm buccolingual width, and 3 mm mesiodistal depth considering 2 mm axial wall depth at the gingival seat of the proximal box. In each group, half of the cavities had their gingival margin of the proximal box placed in enamel 1 mm above the cemento–enamel junction (CEJ) and the other half had their margins placed in dentin 1 mm below the CEJ. The internal line and point angles were rounded and the cavosurface margins were at 90°. The UNC No.15 periodontal probe was used to verify cavity dimensions during cavity preparation and following its completion.

### 2.3. Inlays Fabrication

CEREC Bluecam chair side digital scanner (Sirona, Dentsply, Charlotte, NC, USA) was used to digitally scan the prepared cavities. Then, CEREC MC XL in-office milling machine (Sirona, Charlotte, NC, USA) was used to mill inlays from nanoceramic-reinforced indirect resin composite blocks (Lava Ultimate CAD/CAM, Shade A2, REF no:3312A1-HT, 3M ESPE, St. Paul, MN, USA). Composition of the inlay materials is shown in Table 1.

### 2.4. Study Groups

The 32 teeth were randomly allocated to four groups according to the type of luting resin cement used comprising eight cavities in each group. RC group: inlays luted with RelyX Unicem cement (3M ESPE, St. Paul, MN, USA). RU group: inlays luted with RelyX Ultimate cement (3M ESPE, St. Paul, MN, USA). *e*C group: inlays luted with *e*Cement (Bisco, Schaumburg, IL, USA). VE group: inlays luted with Variolink Esthetic DC cement (Ivoclar Vivadent, Schaan, Liechtenstein). The ceramic inlays were luted by a single operator following manufacturer’s instructions. Luting systems composition and cementation procedure are shown in Table 1 and Table 2, respectively. Each luted restoration was then polished using polishing rubber cup (KENDA, Kanalstrasse, Liechtenstein).

### 2.5. Mounting and Sealing of Teeth

A custom-made mold of rubber silicone was fabricated (ExaFast putty, GC America Inc., Alsip, IL, USA). To mount teeth, the mold was filled with an acrylic resin (Orthodontic Resin, Dentsply/Caulk, Milford, DE, USA) where roots of each tooth were imbedded. Coronally, colored nail polish (Revelon Corp, New York, NY, USA) was applied all around cavity margins at a ∼1 mm distance from the tooth-restoration interface. This prevents the silver nitrate from penetrating the crown surface from areas other than the tooth-restoration interface.

### 2.6. Silver Nitrate (AgNO_3_) Staining

Samples were immersed in a radiopaque 50 wt% silver nitrate solution (AgNO_3_, 50% *w*/*w*) for 24 h to allow the stain to penetrate into any interfacial gap at the tooth–inlay interface. The ammonical silver nitrate stain was prepared by dissolving 25 g of silver nitrate crystals (Sigma Chemical Co., St. Louis, MO, USA) in 25 mL of distilled water. The black solution was titrated using a concentrated (28%) ammonium hydroxide (Sigma Chemical, St. Louis, MO, USA) until it became clear as ammonium ions complexed the silver into diamine silver ions ([Ag (NH_3_)_2_]^+^). The resultant solution was further diluted to 50 mL with distilled water, yielding a 50 wt% solution (pH = 9.5).

### 2.7. Micro-Computed Tomoghraphy Scan (Micro-CT) and Image Analyses

To reveal the silver nitrate penetrant at the tooth–inlay interface, micro-CT scans were taken for the teeth after they were dipped in AgNO_3_ solution using Skyscan 1172 micro-CT scanner (Bruker, Kartuizersweg 3B, Kontich, Belgium). The scanning parameters used were 100 kV, 100 µA, 830 ms of exposure, angle of rotation = 0.600 degree, 720 projections and two frames per projection. Each sample was scanned for a total time of 2 h and 30 min. For 3D reconstruction of acquired micro-CT images and image visualization, NRecon (ver.1.6.7.2; Skyscan, Kontich, Belgium) and Data Viewer software (version 1.5.2.4 64-bit; Skyscan) (Bruker, Kartuizersweg 3B, Kontich, Belgium) were used, respectively.

### 2.8. Internal and Marginal Gap Volumes Measurement

The marginal gap was defined as the gap at the tooth-inlay interface at the margin of the restoration while the internal gap was defined as the gap at the tooth–inlay interface internally. Segmentation of silver nitrate stain was possible through multi-level thresholding feature in three dimensions. This feature allows differentiation of different density levels and therefore the identification of high density subjects with increased gray levels. The 3D volume in mm^3^ of the AgNO_3_ penetrant at the interface between the inlay restoration and the gingival floor of its corresponding cavity were calculated by CT-analyser software (Bruker, Kartuizersweg 3B, Kontich, Belgium).

### 2.9. Statistical Analyses

IBM SPSS (Version 16.0, SPSS Inc., Chicago, IL, USA) was used to compare internal and marginal gap volumes between the resin cements through one-way analysis of variance (ANOVA) followed by Tukey’s post hoc test to delineate the significant differences between groups at a significance level of *p* < 0.05. Additionally, an independent *t* test was used to evaluate the differences in these volumes between enamel and cementum surfaces for each resin cement. Sample size calculation was based on the study by Carrera et al. [16], where mean and standard deviation of mean microleakage volume (mm^3^) was reported, assuming an alpha error of 0.05.

## 3. Results

**Figure 1:**
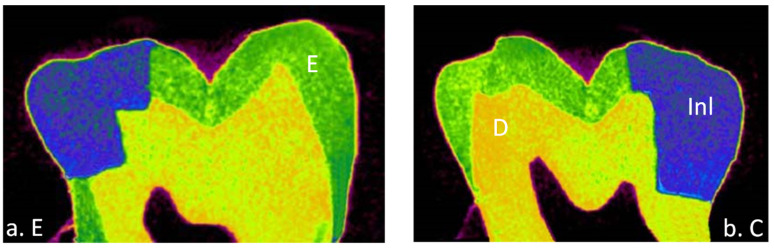
Micro-CT images of internal and marginal gap at the gingival floor of a Class II Lava Ultimate inlay luted with RelyX Unicem resin cement on (**a**) enamel and (**b**) cementum/dentin. Inl, inlay; E, enamel; D, dentin.

**Figure 2:**
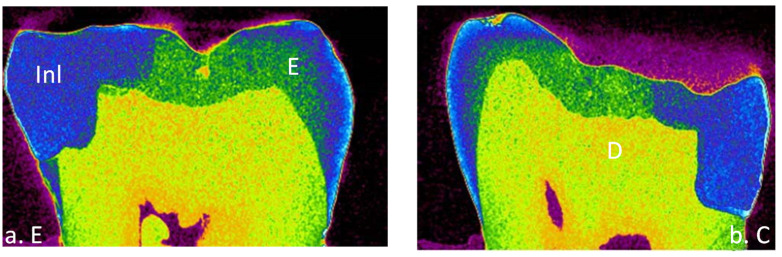
Micro-CT images of internal and marginal gap at the gingival floor of a Class II Lava Ultimate inlay luted with RelyX Ultimate resin cement on (**a**) enamel and (**b**) cementum/dentin. Inl, inlay; E, enamel; D, dentin.

**Figure 3:**
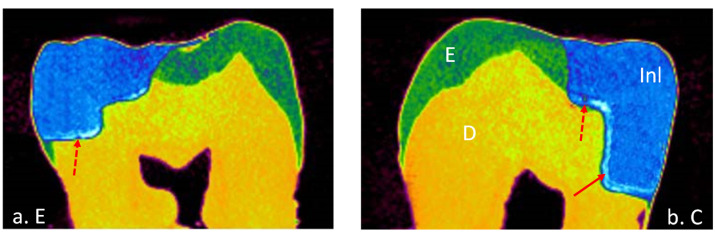
Micro-CT images of internal and marginal gap at the gingival floor of a Class II Lava Ultimate inlay luted with *e*Cement resin cement on (**a**) enamel and (**b**) cementum/dentin. Adhesive layer contains: solid arrow, silver deposits; dotted arrow, void.

**Figure 4:**
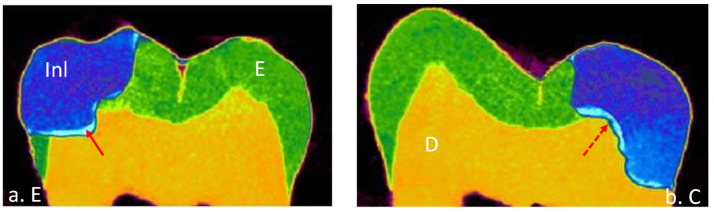
Micro-CT images internal and marginal gap at the gingival floor of a Class II Lava Ultimate inlay luted with Variolink esthetic DC resin cement on (**a**) enamel and (**b**) cementum/dentin. Adhesive layer contains: solid arrow, silver deposits; dotted arrow, void.

A total of 32 teeth were included in this study with 8 teeth in each group of a different cement. The means and standard deviations of the volume of interest represented by internal and marginal gap volumes for each resin cement at enamel and cementum/dentin surfaces are presented in Table 3. All tested groups of the data showed normal distribution with the Shapiro–Wilk test (*p* > 0.05) except RelyX Unicem for internal gap volume (*p* = 0.031). Most of the resin cements expressed homogeneity of variance evaluated by Levene’s test (*p* > 0.05). Thus, parametric analyses were used because the number of samples examined were the same. Internal and marginal gap volume values were the highest for Variolink Esthetic DC at dentin surface (0.629 ± 0.363) and (2.519 ± 1.007), respectively and the lowest for RelyX Unicem at the enamel surface (0.005 ± 0.004) and (0.009 ± 0.003), respectively. Micro-CT images of internal and marginal gaps at the gingival floor of a Class II Lava Ultimate inlay luted with different resin cements are shown in Figure 1, Figure 2, Figure 3 and Figure 4.

### 3.1. Comparison of Internal Gap Volume Associated with Different Types of Resin Cements

One-way ANOVA showed a significant difference in the internal gap volume between the four resin cements groups on enamel (*p* = 0.002), but not for dentin (*p* = 0.078) (Table 3). Multiple comparisons Tukey’s post hoc test showed that the internal gap volume for Variolink Esthetic DC is significantly higher than that for RelyX Unicem (*p* = 0.003) and that for RelyX Ultimate (*p* = 0.004) on enamel surface.

### 3.2. Comparison of Marginal Gap Volume Associated with Different Types of Resin Cements

One-way ANOVA showed a significant difference in the marginal gap volumes between the four resin cements groups on enamel and on cementum (*p* < 0.05) (Table 3). Multiple comparisons Tukey’s post hoc test showed that the marginal gap volume for Variolink Esthetic DC is significantly higher than that for RelyX Unicem on enamel (*p* = 0.006) and cementum (*p* = 0.008) and higher than that for RelyX Ultimate on enamel (*p* < 0.05).

### 3.3. Comparison of Internal and Marginal Gap Volumes on Enamel vs. Cementum Surface

**Figure 5:**
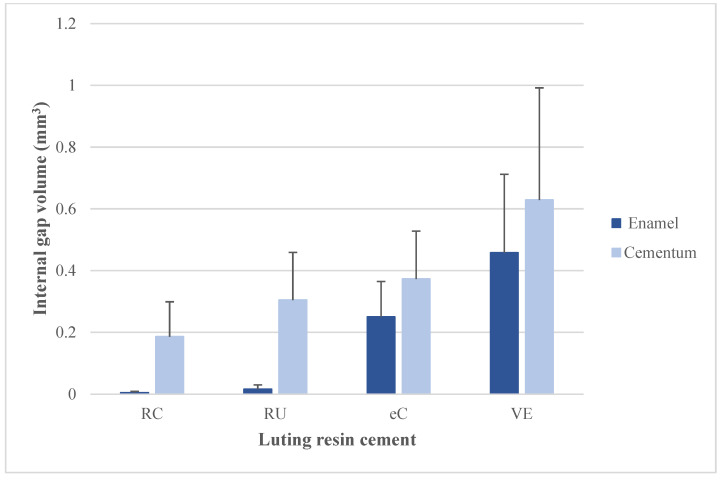
Mean internal gap volume (mm^3^) of inlay restoration associated with different resin cements on enamel vs. cementum/dentin surfaces.

**Figure 6:**
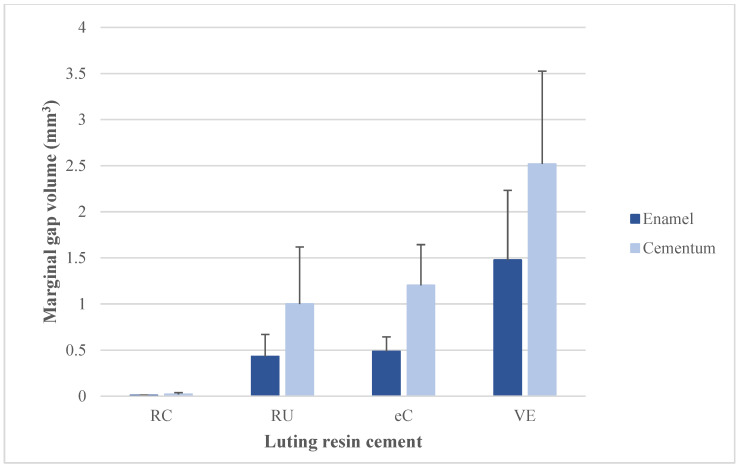
Mean marginal gap volume (mm^3^) of inlay restoration associated with different resin cements on enamel vs. cementum/dentin surfaces.

The t-independent test showed that the internal gap volume is significantly greater on dentin than on enamel for RelyX Unicem, and RelyX Ultimate cements (*p* < 0.05). Whereas, internal gap volume is not significantly different on dentin vs. enamel for *e*Cement (*p* = 0.249) and Variolink Esthetic DC cement (*p* = 0.468) (Table 3 and Figure 5). The *t*-test also showed that the marginal gap volume is significantly greater on cementum than on enamel for *e*Cement (*p* = 0.022). However, marginal gap volume showed no significant differences on enamel vs. cementum for RelyX Unicem, RelyX Ultimate, and Variolink Esthetic DC cements (*p* > 0.05) (Table 3 and Figure 6).

## 4. Discussion

According to the present study results, the null hypotheses that there is no difference in the adaptation of different types of adhesive resin cements and that there is no difference in the adaptation on both enamel and dentin surfaces were rejected. The current investigation addressed one self-adhesive resin cement RelyX Unicem and three universal adhesive resin cements applied in an etch-and-rinse mode; RelyX Ultimate, *e*Cement, and Variolink Esthetic DC with their corresponding universal adhesives; Single-Bond Universal adhesive, All-Bond Universal adhesive and Adhese Universal Viva Pen, respectively. These universal adhesives are intended to provide a durable link between tooth structure, resin cement, and restoration.

The sealing ability of resin cements is vastly dependent on their physical and chemical properties. Their binding ability to other surfaces can be attributed to the presence of acidic monomer 10-methacryloyloxydecyl dihydrogen phosphate (MDP) that may act as a mild self-etching agent to decalcify hydroxyapatite crystals [17]. The MDP monomer phosphate group also forms a chemical bond with calcium ions in the tooth structure and a siloxane bond (Si–O–Si) with the ceramic surface when reacting with ceramic silane coupling agent [18]. Simultaneously, the polymerization of acidic monomer molecules occurs forming a polymer network. This polymerization reaction is responsible for developing the micromechanical retention of the cement to the tooth and is important for the low solubility and durability of the cement [19]. Factors such as the presence of tertiary amines in the resin cement or residual solvent in the adhesive layer are known to interfere with cement polymerization and are detrimental to adhesion and cement adaptation [18]. In this context, all of the included adhesive systems utilize an amine-free initiator and all dual cure cements were subjected to light curing to achieve a higher degree of polymerization of resin monomer than that with chemical curing only and, subsequently, an increased strength and resistance to hydrolysis [20,21]. Nevertheless, the variation in marginal and internal gap volumes found in this study may indicate differences in polymerization shrinkage for different types of resin cements and possible retraction from the adherent surface.

Adaptation of resin cements on the tooth surface can be affected by other factors such as material handling. The cementation process involving multiple steps is technique sensitive. The more technique sensitive the protocol is, the greater the chance for undesirable outcomes, including increased cement thickness, voids, and subsequent marginal gaps. Peroz et al. indicated that the greater marginal seal of RelyX Unicem and Panavia cements is due to less or no multiple steps involved in the luting process [13]. Another investigation reported the lowest percolation percentages and therefore the best sealing ability in association with RelyX Unicem, and RelyX Ultimate cements when compared to eight other different types of cements [22]. The present investigation demonstrates that the adaptation of Lava Ultimate inlay luted with RelyX Unicem cement with no intermediate steps involved in the luting process is higher than the rest of tested cements.

As a self-adhesive resin cement, RelyX Unicem was the only cement used in this study with a distinct method of application. The technique involves no prior etching or priming of tooth structure. This cement of mild initial acidity was suggested not to cause total removal of the smear layer or a significant decalcification and penetration of the cement into the tooth structure to form resin tags inside the dentinal tubules [23]. However, RelyX Unicem exhibits unique phosphate groups in its methacrylate monomer that bind to the calcium of hydroxyapatite crystals of the tooth establishing a stable chemical adhesion [24]. Moreover, as a consequence of this binding of phosphoric acid group to the calcium and to the alkaline fillers present in the cement, neutralization occurs and the cement pH rises rapidly, enhancing the polymerization reaction and preventing cement hydrolysis [12,23].

On the other hand, RelyX Ultimate cement was utilized in this study in conjunction with its Single-Bond Universal adhesive in an etch-and-rinse mode. According to our findings, this cement revealed a significantly higher marginal and internal adaptation than Variolink Esthetic DC cement. An explanation of the good seal of RelyX Ultimate could be that Single-Bond Universal adhesive contains Vitrebond copolymer that promotes adhesion to tooth surface through an interaction between free carboxylic groups and calcium [25]. In addition, according to the manufacturer, the adhesive has a rehydration effect on collapsed collagen fibers after phosphoric acid etching that can enhance the formation of a hybrid layer.

The adaptation of adhesive resin cements is influenced by their viscosity. Cements with low viscosity are cable of flowing better and achieving greater depth of penetration into microretention gaps [26] and forming thin film thickness for superior marginal seals [27]. Zeller studied the influence of temperature on cements viscosity and polymerization reaction. He found great variation in the viscous behavior of different composite resin cements at different temperatures due to differences in their polymerization kinetics [19]. In this in vitro study, the cementation procedure was performed at room temperature and the initial gap formations of composite resin cements were evaluated in a static situation. At the clinical level, however, cementation occurs at a higher temperature of the oral cavity and the marginal seal is challenged by functional stressors. Baldi et al. concluded that cyclic loading increases the interfacial gap opening for overlays cemented on endodontically-treated teeth, particularly if they were not restored with posts [28].

An etch-and-rinse protocol was followed for all the cements used in this study except for the self-etch, self-adhesive RelyX Unicem group. Former studies have shown that a superior gap-free marginal seal for ceramic inlays can be achieved with etch-and-rinse system in comparison to self-etch or self-adhesive cement systems [29,30,31]. Phosphoric acid etching of enamel is known to create micro-porosities in enamel into which the unfilled resin can infiltrate to form a strong micromechanical bond. Similarly, phosphoric acid application on dentin produces decalcification of inorganic matrix and opening of dentinal tubules. However, the micromechanical bonding to dentin is more complicated in nature than enamel due to the heterogenicity of the dentin structure, the presence of a smear layer, dentinal fluid and the probability of collagen fibrils collapse [32,33].

In agreement with several previous studies, our findings concluded that internal and marginal adaptation to enamel was superior than that to dentin [12,34]. In one study, it was reported that microleakage at enamel margin was significantly lower than that at cementum margin of all ceramic MOD inlay restorations cemented with four different self-adhesive resin luting agents [35]. Additionally, another study on the microleakage of two self-adhesive cements revealed that dye penetration at dentin margin was greater than that at enamel margin of the direct composite inlays [36].

The findings of the current study have to be considered in light of some limitations. Since this study is an in vitro study, it is important to be cautious with generalizing its results to clinical settings. Further research to test the marginal and internal gap formation associated with different resin cements for ceramic inlays using larger sample size and under thermodynamic scenarios is needed.

## 5. Conclusions

When cementing resin nanoceramic inlays, clinicians have to consider the type of adhesive resin cement used, as different cements display different marginal and internal gap formation at the inlay–tooth interface. Under the conditions and limitations of the current in vitro study, the following conclusions can be drawn:

1. The internal and marginal adaptation on enamel surface of RelyX Unicem and RelyX Ultimate resin cements were comparable to each other and to *e*Cement but significantly better than Variolink Esthetic DC resin cement.
2. Regardless of the adhesive resin system used, adaptation on enamel surfaces is superior to that on dentin surfaces.

## Figures and Tables

**Table 1:** Materials used.

Material	Manufacturer	Composition
LAVA Ultimate CAD/CAM blocks	3M ESPE	Polymer (20 wt%; Bis-GMA, UDMA, Bis-EMA, and TEGDMA (20 wt%), nanofiller of SiO_2_ of 20 nm and ZrO_2_ of 4–11 nm (80 wt%), nanocluster particles derived from the nanomers (0.6–10 μm) and silane coupling agent.
Acid etchant	3M ESPE	35% phosphoric acid
RelyX Unicem (RC)	3M ESPE	Powder: glass fillers, silica, calcium hydroxide, self-cure initiators, pigments, light-cure initiators.Liquid: methacrylated phosphoric esters, dimethacrylates, acetate, stabilizers, self-cure initiators.
Single Bond Universal	3M ESPE	Bis-GMA, HEMA, decamethylene dimethacrylate, ethanol, water, silane-treated silica, 2-propenoic acid, methacrylated phosphoric acid, copolymer of acrylic and itaconic acid, ethyl-4-dimethylaminobenzoate, silane, CQ, MEK, (dimethylamino)ethyl methacrylate.
RelyX Ultimate Clicker Dual Cure (RU)	3M ESPE	Base paste: Methacrylate monomers, radiopaque, silanized fillers, initiator, stabilizers, rheologic additives.Catalyst paste: Methacrylate monomers, radiopaque alkaline fillers, initiator components, stabilizers, pigments, rheologic additives, fluorescence dye, dual-cure activator (chemically curing activator for scotchbond universal adhesive)
ALL-BOND Universal	Bisco	Bisphenol A diglycidylmethacrylate (20–50%), ethanol (30–50%), MDP (5–25%), 2-hydroxyethyl methacrylate (5–25%)
*e*Cement (*e*C)	Bisco	Base paste: Ytterbium fluoride (10–20%), bisphenol A diglycidylmethacrylate (10–30%), UMDA (10–30%), ytterbium oxide-silica (1–5%), tetrahydrofurfuryl methacrylate (1–5%), TMPTMA (1–5%)Catalyst paste: Bisphenol A diglycidyl-methacrylate 10–30%, dibenzoyl peroxide, technically pure < 1%
Adhese Universal VivaPen	Ivoclar Vivadent	Methacrylates: MDP, MCAP, HEMA, Bis-GMA, D_3_MA, water, ethanol, highly dispersed silicon dioxide, initiators and stabilizers
Variolink Esthetic DC (VE)	Ivoclar Vivadent	UMDA, methacrylate monomers as 1,10-decandiol dimethacrylate, a-dimethylbenzyl hydroperoxide, initiators, stabilizers, pigments and inorganic fillers of ytterbium trifluoride, spheroid mixed oxide (particle size: 0.04–0.2 μm. Mean particle size: 0.1 μm & 67 wt% = 38 vol%)

MDP, methacryloyloxydecyl dihydrogen phosphate; MCAP, methacrylated carboxylic acid polymer; HEMA, hydroxyethyl methacrylate; Bis-GMA, bisphenol A glycidyl methacrylate; D_3_MA, decandiol dimethacrylate; TMPTMA, trimethylolpropane trimethacrylate; CQ, camphorquinone; MEK, methyl ethyl ketone.

**Table 2:** Cementation procedures.

Study Group	Cementation Procedure
RC group	Mechanically mix the capsule (Capmix) for 15 s Apply RelyX Unicem cement using tip directly to the fitting surface of inlay, seat inlay and maintain under finger pressure for 2 min Remove excess cement Light cure * for 40 s
RU group	Total etch for 15 s Water rinse for 15 s Dry with cotton pellet Apply Single Bond Universal adhesive to tooth surface for 20 s Gently air dry for 5 s Light cure * for 10 s Dispense equal quantities of RelyX Ultimate cement pastes and mix with spatula for 20 s Apply cement onto fitting surface of inlay Seat inlay and maintain under finger pressure for 2 min Remove excess cement Light cure * for 40 s
*e*C group	Total etch for 15 s Water rinse for 15 s Dry with cotton pellet Apply two coats of All-BOND Universal adhesive to tooth surface for 20 s Thoroughly air dry for 10 s Light cure * for 10 s Apply *e*Cement through auto-mix tip onto fitting surface of the inlay Seat inlay and maintain under finger pressure for 2 min Remove excess cement Light cure * for 40 s
VE group	Total etch for 15 s Water rinse for 15 s Dry with cotton pellet Apply Adhese Universal adhesive to tooth surface for 20 s Thoroughly air dry for 10 s Light cure * for 10 s Apply Variolink Esthetic DC cement through auto-mix tip onto fitting surface of the inlay Seat inlay and maintain under finger pressure for 2 min Remove excess cement Light cure * for 40 s

* A calibrated LED light cure (Bluephase 20i, Ivoclar/Vivadent AG, Schaan, Liechtenstein, Austria) was used to polymerize at 1200 mW/cm^2^.

**Table 3:** Mean (SD) of internal and marginal gap volumes in mm^3^ for different resin cements on enamel vs. cementum/dentin surfaces.

Resin Cement	Internal Gap Volume (mm^3^)		Marginal Gap Volume (mm^3^)	
	Enamel	Dentin	Enamel	Cementum
RelyX Unicem	0.005 (0.004) ^a^	0.186 (0.113) ^a^	0.009 (0.003) ^a^	0.021 (0.018) ^a^
RelyX Ultimate	0.016 (0.014) ^a^	0.305 (0.154) ^a^	0.432 (0.239) ^a^	1.000 (0.619) ^a,b^
*e*Cement	0.250 (0.115) ^a,b^	0.373 (0.155) ^a^	0.487 (0.157) ^a,b^	1.203 (0.441) ^a,b^
Variolink Esthetic DC	0.458 (0.254) ^b^	0.629 (0.363) ^a^	1.478 (0.755) ^b^	2.519 (1.007) ^b^

Superscript letters indicate homogenous subsets (within which *p* > 0.05) where comparison has been made with respect to resin cements at each enamel and dentin surface.
